# The influence of travel time on perceived traveled distance varies by spatiotemporal scale

**DOI:** 10.1007/s00221-024-06880-1

**Published:** 2024-07-02

**Authors:** Cindy Jagorska, Martin Riemer

**Affiliations:** 1 Biological Psychology and Neuroergonomics, Technical University Berlin, 10623 Berlin, Germany; 2 Bernstein Center for Computational Neuroscience (BCCN), Philippstraße 13, 10115 Berlin, Germany; 3 Center for Behavioral Brain Sciences (CBBS), Magdeburg, Germany

**Keywords:** Path integration, Time–space interference, Virtual reality, Distance perception, Optic flow

## Abstract

The influence of travel time on perceived traveled distance has often been studied, but the results are inconsistent regarding the relationship between the two magnitudes. We argue that this is due to differences in the lengths of investigated travel distances and hypothesize that the influence of travel time differs for rather short compared to rather long traveled distances. We tested this hypothesis in a virtual environment presented on a desktop as well as through a head-mounted display. Our results show that, for longer distances, more travel time leads to longer perceived distance, while we do not find an influence of travel time on shorter distances. The presentation through an HMD vs. desktop only influenced distance judgments in the short distance condition. These results are in line with the idea that the influence of travel time varies by the length of the traveled distance, and provide insights on the question of how distance perception in path integration studies is affected by travel time, thereby resolving inconsistencies reported in previous studies.

## Introduction

Path integration is the ability to integrate linear and angular self-motion to track ones’ location over time and space (McNaughton et al. 2006). This includes the ability to judge the length of traveled distances via self-motion (Robinson and Wiener 2021) and enables spatial updating of landmarks (Müller et al. 2018; Wolbers et al. 2008). These traveled distance judgments can be influenced by different factors, one being the required travel time. However, previous studies revealed inconsistent findings with respect to the perceptual relationship between travel time and traveled distance. When comparing walking and running a fixed distance on campus, Cohen et al. (1963) found that the same traveled distance of about 45m appeared longer when the associated travel time was longer. Such a positive relationship between travel time and perceived traveled distance has also been reported in other studies using either real world set-ups (Herman et al. 1983) or visual optic flow in virtual desktop environments (Ellmore and McNaughton 2004; Harris et al. 2012; Redlick et al. 2001). This positive relationship seems intuitive, since covering a larger distance usually requires more time. However, there are other studies, both conducted in real and virtual (predominantly) desktop environments, reporting no (Herman et al. 1984; Lederman et al. 1987; Lappe et al. 2007; Riemer et al. 2018) or even a negative relationship between travel time and perceived traveled distance, that is, longer travel times leading to a relative underestimation of traveled distances (Frenz and Lappe 2005; Riemer et al. 2022).

In the present study we propose an explanation for these inconsistent findings, based on the idea that the influence of travel time on perceived traveled distance differs for small compared to larger spatiotemporal scales. We argue that for longer distances, traveled distance judgments are based on travel time, while for shorter distances, they are mainly based on movement speed.

The diverse interconnection of space and time at large versus small spatiotemporal scales is already evident in our everyday language. While it is common to state that the nearest train station is 10 min away (equating the distance with the time it takes to cover it), it would be atypical to say that the window one just opened is 3 s away. The idea that inconsistencies in the literature concerning the influence of travel time on perceived distances occur due to differences in spatiotemporal scales has already been described by Montello (1997), who argues that a positive influence of travel time on perceived traveled distance might be restricted to large spatiotemporal scales. However, Montello (1997) also points out that it is unclear where a “large” spatiotemporal scale begins, and that, by geographic standards, most experimental studies deal with small spatiotemporal scales. The influence of spatiotemporal scale on distance judgments can nevertheless be demonstrated by a relative rather than absolute definition.

Regarding the question of why a positive influence of travel time on perceived traveled distance would not only be absent, but occasionally is even negative, we argue that this is due to the influence of movement speed. Traveled distance is the product of travel time and movement speed, and the perception of both magnitudes is required for an adequate estimation of traveled distance. The inconsistent findings with respect to the direction of the effect that travel time has on the estimation of traveled distance could be reconciled by the assumption that distance judgments are sometimes rather based on movement speed than on travel time, especially when the judged distances and associated travel times are rather short.

For a given distance (e.g., 10m), the associated movement speeds and travel times are diametrically opposed. Increasing speed necessarily leads to a decrease in travel time. Thus, when movement speed serves as a proxy for traveled distance, in the sense that higher speeds indicate longer distances, then travel time and perceived traveled distance would display a negative relationship.

We align our argument with the perspective of Montello (1997), that on larger spatiotemporal scales, such as when covering relatively large distances (e.g., walking to the supermarket), travel time becomes a more influential factor in estimating one’s traveled distance. In contrast, when examining smaller spatiotemporal scales, such as walking a short distance (e.g., crossing a room to open a window), movement speed is often a very salient predictor for the estimation of traveled distance. Consistent with this assumption, a positive influence of travel time on perceived traveled distance is often found for relatively long travel distances up to 45m (Cohen et al. 1963; Ellmore and McNaughton 2004; Harris et al. 2012; Herman et al. 1983; Redlick et al. 2001), whereas a negative influence of travel time on perceived traveled distance was reported for shorter travel distances (up to 3m, Frenz and Lappe 2005) or shorter travel times (up to 4.8s, Riemer et al. 2022). The results of Redlick et al. (2001) show that the positive relation between travel time and perceived traveled distance becomes more prominent with distances increasing from 4 to 32 m.

Previous studies pointed out that egocentric distance estimations can be influenced by the medium of presentation (El Jamiy and Marsh 2019; Kelly 2022). With regards to head-mounted displays (HMDs), Kelly (2022) does report a general compression of distance perception compared to the real-word, but this effect becomes weaker with higher quality HMDs. El Jamiy and Marsh (2019) point to the importance of binocular disparity, which refers to the discrepancy between the two retinal image projections of a specific point in space (Qian 1997). According to El Jamiy et al. (2019), binocular disparity is especially important for the judgment of short distances. Previous work on distance estimation, and especially the studies finding a negative influence of travel time on perceived traveled distance, have utilized desktop tasks that do not allow for binocular disparity. To assess a potential difference in egocentric travel distance estimation between the presentation on a desktop and through an HMD, we incorporated both presentation modes in our study.

The idea that the influence of travel time on perceived traveled distance depends on the spatiotemporal scale has the potential to reconcile inconsistent findings in the literature, because it can explain why some studies did find an effect of travel time and some did not. To test our hypothesis, we implemented a distance production task and directly compared the influence of movement speed on judgments of shorter and longer traveled distances. We hypothesized that, when the to-be-judged travel distances are relatively short, traveled distance judgments are primarily based on movement speed, but when the to-be-judged travel distances are relatively long, traveled distance judgments are primarily based on travel time. Accordingly, we expected that short traveled distances are judged as longer for high versus low movement speed, whereas long traveled distances are judged as shorter when they are combined with high versus low movement speed. To account for potential influences of the presentation mode, we presented our task both on a desktop as well as through an HMD.

## Methods

### Participants

52 adults participated in the study. They were recruited through the TU Berlin portal SONA and compensated either financially (12 euros) or by course credits. All participants gave written informed consent to the experimental protocol, which was in line with the ethical standards of the TU Berlin (protocol number: MR_01_20200323). All participants reported normal or corrected to normal vision. Three participants were excluded because they had problems understanding the task. The final sample consisted of 49 participants (26 male, 22 female, 1 other; mean age = 29.20).

### Experimental task and stimuli

We used a distance production task, in which participants had to produce the egocentrically perceived distance to a previously shown target by stopping a passive simulated forward movement. The task was presented on a PC display (desktop condition) or via a head-mounted display (HMD condition). To ensure a comparable experience of optic flow, the viewpoint in the HMD condition was fixed. Whether participants started with the HMD or desktop condition was counterbalanced. Eye height was set to 1.8m.

The FOV of the virtual environment was the same in both conditions, though it should be noted that the subjects’ FOV was larger in the HMD condition, due to the nature of the HMD compared to a desktop.

**Fig. 1:**
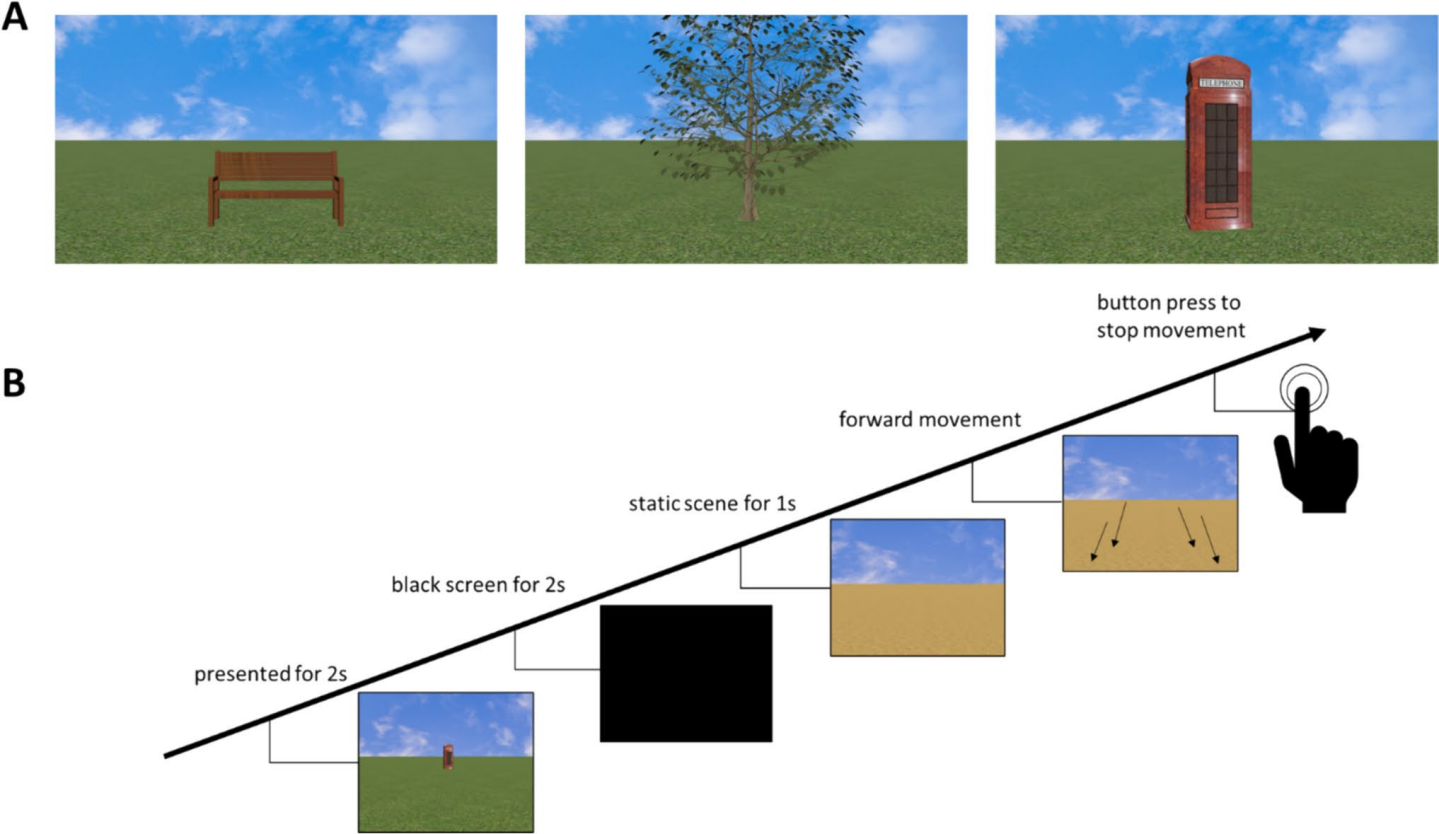
**A** Target objects. **B** Exemplary trial structure

In each trial, participants were presented with a target object (either a phone box, a tree, or a bench; cf. Fig. 1A) on a virtual lawn. The target object was presented for 2s at a distance of either 7 virtual meters (vm) (short distance condition) or 35 vm (long distance condition).^1^ Then, the display turned black for 2s. Afterwards the ground changed to either concrete, sand, or soil, in order to make it harder to just fixate on a point on the ground where the object had been located. After 1s, a straight, simulated forward movement began at a constant speed of either 1.75m/s (low speed condition) or 3.5m/s (high speed condition). The participants were asked to press the space bar once they believed they reached the point where the previously seen object was located. Target objects and trial structure can be seen in Fig. 1. Both the desktop and the HMD condition consisted of 72 trials, leading to a total of 144 trials. After the completion of 36 trials participants could take a short break. Within every 36 trials the order of the distance and speed conditions was randomized. At the end of the desktop and HMD condition participants received feedback regarding their performance. The task was programmed in Vizard (v5.0, WorldViz). The desktop condition was presented on a computer screen (59.5cm × 33.5cm) with participants being seating approximately 65cm away from the desktop. The HMD condition was presented on the HTC Vive Pro. The whole experiment lasted for about 60 min.

### Statistical analysis

#### Behavioral data

For the analysis, the ratio between the produced distance and the target distance was used as the dependent variable. Values above 1 indicate an overproduction, values below 1 indicate an underproduction. Note that we were not interested in these objective over- and underproductions per se, but in the relative over- and underproductions between conditions.^2^ The data were analyzed in R, which was also used to generate figures (R Core Team 2016), using a linear mixed effect model (R package lme4 1.1–27.1; Bates et al. 2014). We fitted a 2 × 2 × 2 model design, including distance (long vs. short), speed (low vs. high) and presentation mode (desktop vs. HMD). Random intercepts (but not random slopes) were included to account for the variability among subjects. Outliers were defined by the median absolute deviation (MAD) procedure (Leys et al. 2013). Outliers were defined within participants and all conditions. We used the R package *Routliers*, and a threshold of 3, which, according to Leys et al. (2013) is considered conservative. A total of 0.03% of data points were excluded.

The data and analysis script can be found at https://github.com/cindyjag/TimeSpeedSpatiotemporalScale.

### Subjective ratings

After the experiment, participants were asked to fill out subjective ratings concerning their experience during the experiment. We were especially interested in the participants’ reports about whether they rather utilized time or speed for their distance judgments. The question that assessed this was “For the estimation of the short/long distances I rather used…”. Participants could then choose to tick on a 20-point Likert-Scale with “Time” on the left extreme and “Speed” on the right extreme. The questions were asked separately for both presentation modes. Due to the non-assumption of interval scaled answers, we employed paired Wilcoxon signed-rank tests to examine differences in these ratings.

## Results

### Behavioral results

**Fig. 2:**
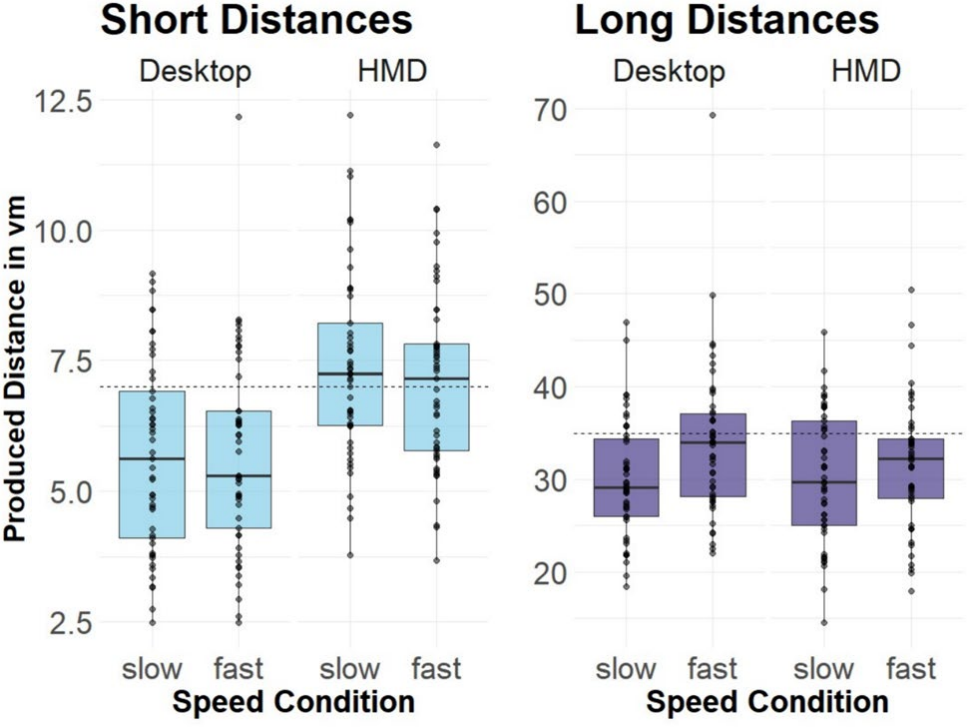
Mean produced distances in the short distance condition (left) and the long distance condition (right) by presentation mode and speed condition

**Fig. 3:**
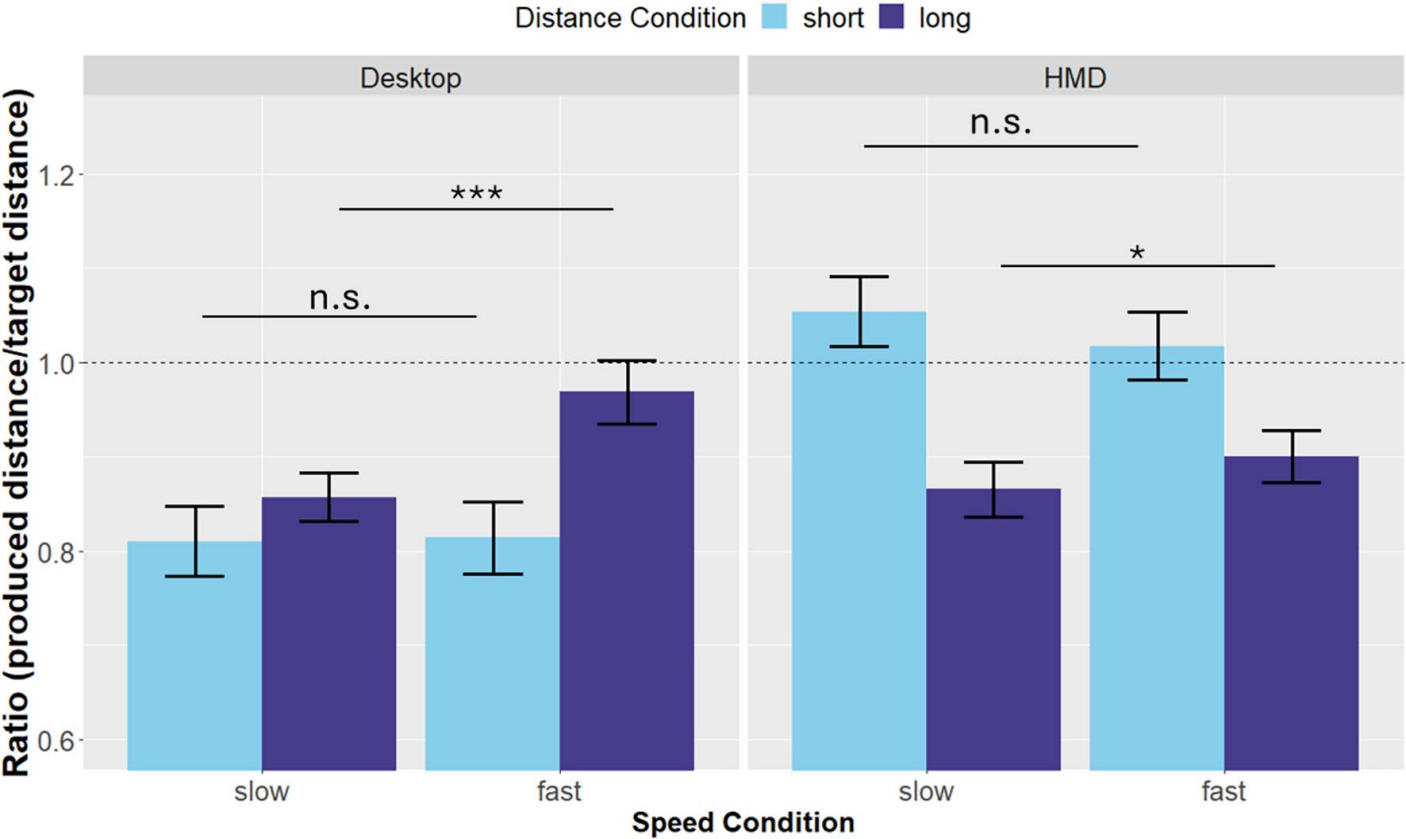
Mean ratios between produced distances and target distances by speed condition, presentation mode and distance condition. Higher values indicate higher produced distances. Error bars represent the mean ± the standard error. For long distances, higher speed led to longer distance productions. This effect was found across presentation modes and was absent for short distances

Produced distances are depicted in Fig. 2. As can be seen, the target distance as well as the presentation mode influenced the participants’ produced distances. In Fig. 3 it can be seen that there is a difference in the impact of speed by distance condition. We did not find a main effect of distance (*β* = 0.05, SE = 0.03, *p* = 0.182) or speed (*β* = 0.00, SE = 0.034, *p* = 0.914), but a main effect of presentation mode (*β* = 0.24, SE = 0.04, *p* < 0.001). There was a significant interaction between distance and speed (*β* = 0.11, SE = 0.04, *p* = 0.029), see Fig. 3, indicating that for long distances, higher speed resulted in relative overproduction, whereas this was not the case for short distances. These results are in line with our hypothesis that judgments of longer distances are influenced by travel time, and hence a lower speed should lead to higher distance estimates (note that *over*production is equivalent to *under*estimation). T-tests revealed a significant difference for long distances (*t*(97) =  − 6.16, *p* < 0.001), but not for short distances (*t*(97) = 0.94, *p* = 0.35). The significant interaction between distance and presentation mode (*β* =  − 0.22, SE = 0.49, *p* < 0.001), see Fig. 3, points to the short distance condition being more influenced by presentation mode compared to the long distance condition. This was further confirmed by t-tests, revealing a significant effect of presentation mode for short distances (*t*(97) = 9.95, *p* < 0.001), whereas no significant difference was observed for long distances (*t*(97) =  − 1.59, *p* = 0.12). We neither found an interaction between speed and presentation (*β* = − 0.04, SE = 0.04, *p* = 0.24) nor between speed, presentation, and distance (*β* =  − 0.03, SE = 0.06, *p* = 0.82).

**Fig. 4:**
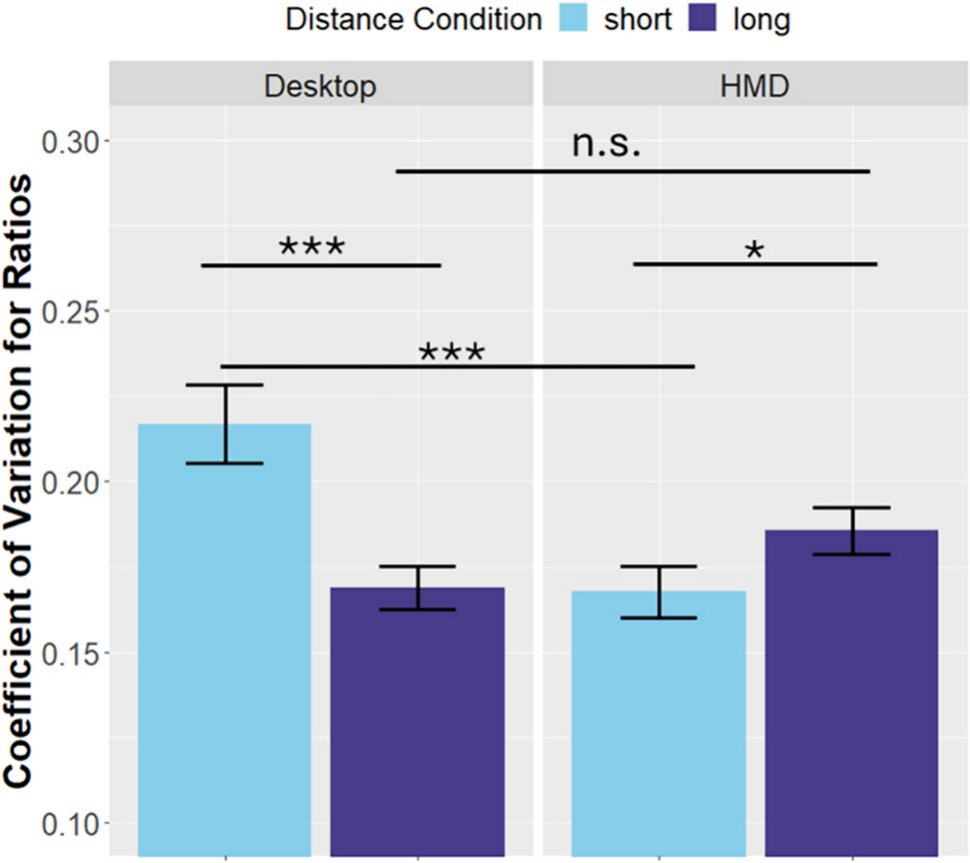
Mean coefficient of variation (CV) regarding the ratios by presentation mode and distance condition (averaged over speed conditions). Error bars represent the mean ± the standard error. The CV was especially high for short distances in the desktop condition

To further analyze the data, we additionally performed t-tests on the coefficient of variation for the aggregated ratios. The results are visualized in Fig. 4. Interestingly, the coefficient of variation was especially high in the short-distance desktop condition. The t-tests revealed that, after correction, there was a significant difference between the short and long distance condition in both the HMD condition (*t*(97) = 2.48, *p* = 0.014), and the desktop condition (*t*(97) = 5.17, *p* < 0.001). While it is plausible that the short distances generally lead to more variation in productions, the coefficient of variation was also higher in the short distance desktop condition than in the short distance HMD condition (*t*(97) = 5.21, *p* < 0.001). This was not the case between the long distance desktop condition and the long distance HMD condition (*t*(97) = − 2.23, *p* = 0.02).

### Subjective ratings

**Fig. 5:**
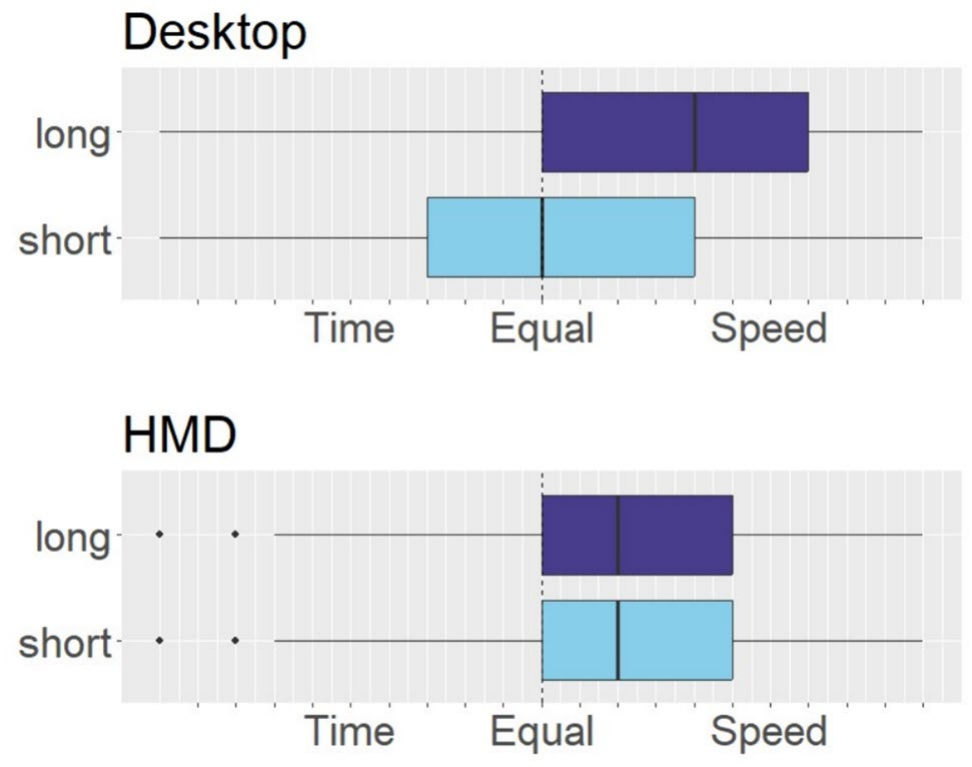
Self-reported usage of time and speed for distance judgments by distance conditions. The black lines represent the medians, the boxes themselves contain the interquartile range, encapsulating 50 percent of the data. The whiskers extend from the first quartile minus 1.5 times the interquartile range to the third quartile plus 1.5 times the interquartile range

Wilcoxon signed-rank tests showed that, with respect to the subjective reports about the utilization of travel time and movement speed as a predictor of traveled distance (see Fig. 5), for the desktop version, participants reported focusing significantly more on speed in the short distance condition compared to the long distance condition (V_48_ = 333, *p* = 0.04). We did not find this for the HMD condition (V_48_ = 229, *p* = 0.3).

## Discussion

In the present study, we investigated the influence of travel time and movement speed on perceived traveled distance in virtual environments. Our aim was to reconcile inconsistent findings of previous studies with respect to the relation between travel time and perceived traveled distance. We argued that these inconsistencies could be explained by differences in the length of traveled distances.

Our hypothesis was that long distances are judged as shorter when they are traveled at a high versus a low speed (resulting in a positive relationship between travel time and perceived traveled distance). This was based on the notion that studies that do find a positive relationship between travel time and perceived traveled distance, though greatly varying in the methods employed, usually use relatively long distances. For example, in real-world studies by Cohen et al. (1963) and Herman et al. (1983), distances ranging from 10 to 45m were used. In a desktop optic flow task, Ellmore and McNaughton (2004) used distances over 16m. All these studies found a positive influence of travel time on perceived traveled distance.

We additionally hypothesized that short distances might be judged as longer when they are traveled at a high speed (resulting in a negative relationship between travel time and perceived traveled distance). This hypothesis was derived from the fact that the studies that do report a negative influence of travel time on perceived traveled distance used rather short distances (up to 3m, Frenz and Lappe 2005) or, as in the study by Riemer et al. (2022), short travel times (up to 4.8s). Riemer et al. (2022) argued that the negative effect of travel time on perceived distance might result from the mediating influence of speed.

The results confirm our hypothesis that the effect of travel time on perceived traveled distance differs for relatively long versus relatively short traveled distances. Direct comparisons, separate for long and short traveled distances, showed that this effect was primarily based on the judgments of long traveled distances, for which travel time showed a positive influence on perceived traveled distance. When movement speed was low (and hence travel time was relatively long), participants produced shorter distances compared to the high movement speed condition. This points to a relative overestimation of traveled distance in the low speed condition compared to the high speed condition (because production and estimation tasks are negatively correlated, see Mitchell and Davis 1987; Riemer et al. 2014). In contrast, a direct comparison of the two speed conditions for the short traveled distances did not reveal a significant difference.

When asked about their strategy to solve the task, most participants reported fixating a point on the ground and following it until arrival, which was described as easier in the short distance condition. Since fixating the ground is essential for the processing of visual optic flow (Banton et al. 2005; Bremmer and Lappe 1999) and optic flow was (in our design) the only available cue giving rise to the experience of speed, this is in line with the idea that the influence of movement speed was more pronounced for short distances. Accordingly, participants reported placing more emphasis on movement speed for short distances, aligning with the assumption that speed is more salient and might be a more important predictor for short distances. It is, however, also possible that the strategy of “fixating a point on the ground” inhibited speed-based strategies, that is, participants might just have kept fixating one point and stopped the movement when that point is reached. Although this would not prevent the processing of movement speed and travel time, it would remove the necessity to account for this information when judging the traveled distance and ultimately reduce the influence of speed (and thus travel time). According to this interpretation, the observation that the perception of short (as compared to long) distances was not influenced by speed might indicate that short distances were not judged on the basis of path integration processes. This issue needs further examination in future studies. Restricting the vertical field of view (e.g., looking down) and/or eye-tracking has been successfully employed to specify how different gaze patterns contribute to the perception of optic flow (e.g., Banton et al. 2005; Ellmore and McNaughton 2004), demonstrating that these methods can also be useful in future research to test whether the effect of movement speed on distance judgments is modulated by specific gaze patterns.

Evidence for the usage of movement speed as an indicator of traveled distance can be found in neuroimaging studies, reporting the involvement of speed-sensitive brain regions in distance judgments (Indovina et al. 2016; Riemer et al. 2022; Wolbers et al. 2007). In the study by Riemer et al. (2022), showing a significant negative influence of travel time on perceived traveled distance, this negative interference effect was associated with higher representational similarity in posterior parietal areas, which are known for their involvement in speed processing (Britten 2008; Martinez-Trujillo et al. 2007). The task employed in Riemer et al. (2022) involved short travel times (< 5s), underscoring the idea that, on a small spatiotemporal scale, movement speed is a more plausible predictor of traveled distance. In a similar vein, Stangl et al. (2020) investigated different sources of errors in path integration and found that judgments of traveled distances are mainly affected by a biased estimation of self-motion speed. As relatively short distances were tested in their study (approximately 2–6 m), this finding is in line with the hypothesis presented here.

Previous work that was done on the interrelation between travel time and travel distance (Cona et al. 2021; Gladhill et al. 2022, 2024; Robinson and Wiener 2021) demonstrates the mutual influence between travel time and traveled distance, behaviorally as well as with regard to shared neuronal processes. The mentioned studies typically encompass travel time and traveled distance reproduction tasks, where participants are first presented with an egocentric forward motion of which they will then have to either reproduce the traveled distance or travel time. To prevent participants from using strategies such as counting, these studies often vary the movement speed between the presentation and reproduction phase. This provides an elegant solution to investigate mutual influences in judgments between travel time and traveled distance. Studies on these interrelations usually demonstrate that travel time and travel distance judgments are related. But these studies typically use movement speed as a way to decouple travel time from travel distance, rather than to systematically investigate its influence. Regarding the interactions between time and distance, the present study demonstrates that the influence of travel time on perceived traveled distance depends on the spatiotemporal scale.

The productions we see in the short distance desktop condition are somewhat unexpected. While a tendency to the mean of the used stimuli can often be seen in this type of task (Petzschner and Glasauer 2011), and can be seen in the HMD condition, this is not true for the productions in the desktop condition, where we see a similar pattern for short and long distances (see Figs. 2 and 3). Additionally, the coefficient of variation is especially high for the short distance desktop trials (see Fig. 4). A possible explanation is that the short target distances were perceived differently in the desktop vs. HMD condition. El Jamiy et al. (2019) point out that depth-cues in form of binocular disparity are important for the correct estimation of short distances, while they are not as important for the correct judgment of longer distances. Participants were only able to use binocular depth-cues in the HMD condition, which would explain that the difference in productions between desktop and HMD presentation is more pronounced for short distances. This explanation is in line with the fact that participants produced the distance to the target most accurately in the short distance HMD condition (see Fig. 3). Another possible explanation for this observation stems from the difference in the field of view between the conditions. Movement speed is perceived higher when a larger field of view is available (Pretto et al. 2009). In line with the idea that movement speed is more salient in the short distance condition, it seems plausible that increases in perceived movement speed would be reflected in a more pronounced influence on the short distances.

In summary, our results show that the influence of travel time on perceived traveled distance differs depending on the length of the distances to-be-judged. For long distances, more travel time leads to higher traveled distance estimations. This is not the case for short distances. Accordingly, we argue that the inconsistent findings in the literature regarding the influence of travel time on perceived traveled distance can be explained by the length of the distances used, because their estimations rely on different estimators. Long distances are judged primarily on the basis of travel time, whereas short distances are rather judged on the basis of movement speed. This explanation is in line with earlier ideas that an influence of travel time on perceived traveled distance would show up more strongly in larger spatiotemporal scales (Montello 1997).

An area for future investigation lies in the intrinsic connection between traveled distance and travel time in this study. While it aligns with the common association of these two magnitudes in everyday life, it poses a challenge in attributing the observed differences in distance judgments specifically to variations in either travel time or traveled distances. In other words, the present study leaves it undecided whether the different weighting of the indicators (i.e., travel time or movement speed) are due to the length of the to-be-judged distance or the duration of the associated travel time. To address this question, future research should explore these possibilities by comparing conditions in which the same distances are covered within different travel times.

## Conclusion

Our results confirm that the influence of travel time on perceived traveled distance varies for longer as compared to shorter distances. For longer distances, there is a positive association between travel time and perceived traveled distance, whereas this relationship is not evident for shorter distances. The presentation mode only impacted distance judgments in the short distance condition. Our finding offers a potential explanation for inconsistent findings with respect to the relation between actual travel time and perceived traveled distance.

## Data Availability

The data and analysis script can be found at https://github.com/cindyjag/TimeSpeedSpatiotemporalScale.

## Footnotes

1 The objects were chosen to be of a well-known size for many people. The distances were chosen on the notion that the objects could be seen well in the short and long distance condition.

2 Also, note that an overproduction points to an underestimation of the perceived traveled distance (at the point when the correct distance is covered, the participant underestimates the distance already traveled and therefore continues walking), while an underproduction indicates an overestimation of the perceived traveled distance (Bindra and Waksberg 1956; Mitchell and Davis 1987; Riemer et al. 2014).
